# Supplementary material for: Corin Deficiency Diminishes Intestinal Sodium Excretion in Mice
Source: Biology (Basel). 2023 Jul 1;12(7):945. doi: 10.3390/biology12070945 (PMC10376046; doi:10.3390/biology12070945)
Supplement: Supplementary file 1 [file biology-12-00945-s001.zip › biology-2474906-supplementary.pdf]

Corin Deficiency Diminishes Intestinal Sodium and Chloride Excretion in Mice

Supplementary Figures S1-S4 and Table S1

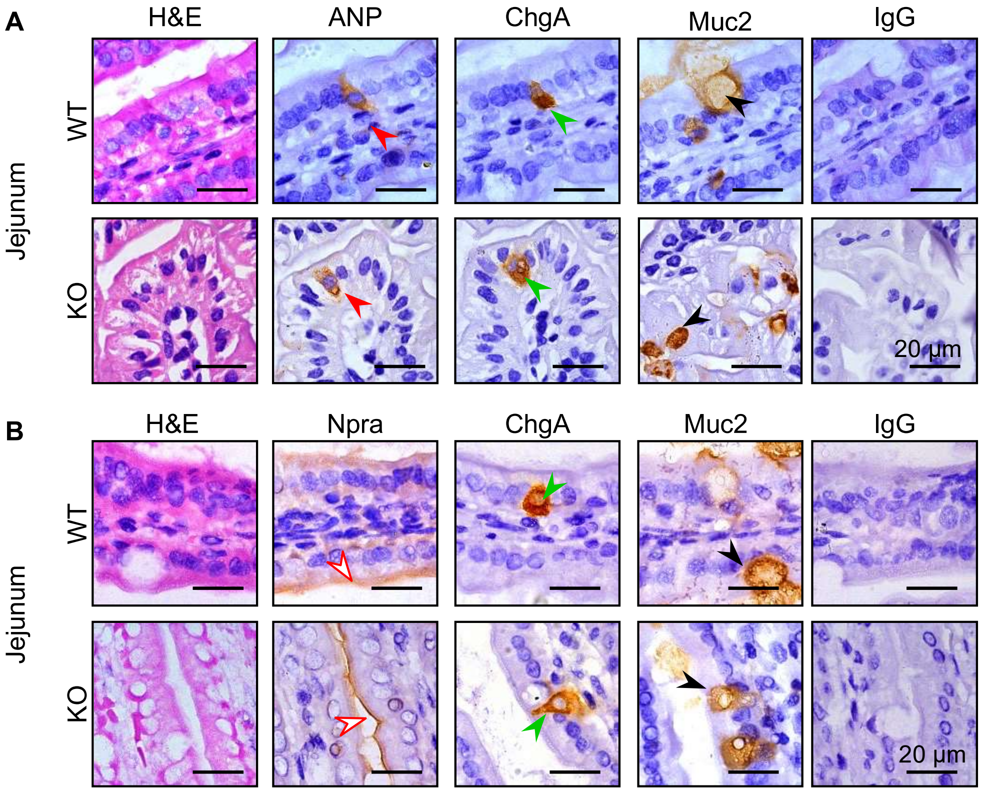

Supplementary Figure S1. ANP and Npra protein expression in jejunal sections from WT and *Corin* KO mice. Immunohistochemical staining of pro-ANP/ANP (ANP) (**A**) (filled red arrowheads) and Npra (**B**) (open red arrowheads) in jejunal sections form WT and *Corin* KO mice (male, 10-12 weeks old on normal-salt diet). ChgA (green arrowheads) and Muc2 (black arrowheads) staining was included as controls. As a negative control, a normal IgG was used instead of the primary antibody. Scale bars: 20 μm. Data are representative of at least three experiments in each set of the experiments.

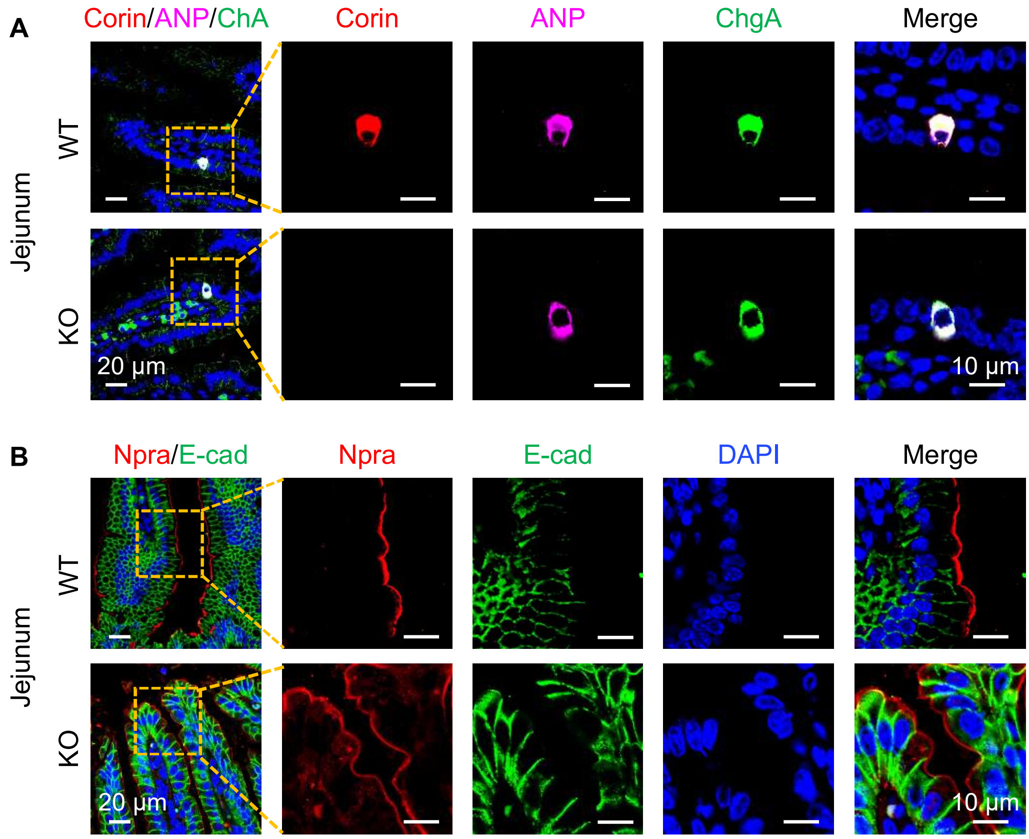

Supplementary Figure S2. Corin, ANP, and Npra protein expression in the mouse jejunum. Co-immunofluorescent staining was performed in jejunal sections from WT and *Corin* KO mice (male, 10-12 weeks old on normal-salt diet). (**A**) Co-staining of Corin (red), ANP (purple), and ChgA (green) in enteroendocrine cells of jejunal sections from WT (top) and *Corin* KO (bottom) mice. (**B**) Co-staining of Npra (red) and E-cad (green) on the luminal surface in jejunal sections from WT (top) and *Corin* KO (bottom) mice. DAPI was used to stain cell nuclei (blue). Scale bars are indicated. Data are representative of at least three experiments in each set of the experiments.

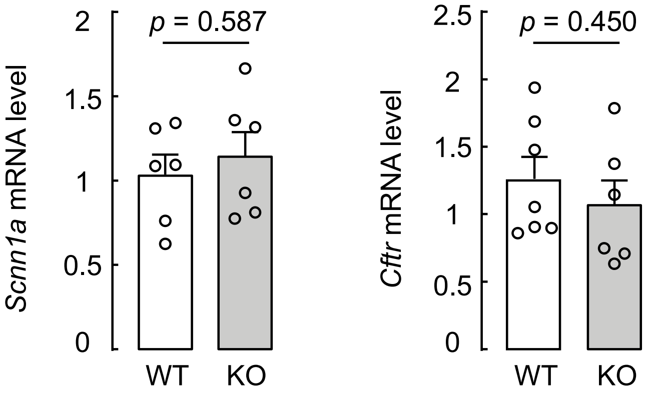

Supplementary Figure S3. *Scnn1a* and *Cftr* mRNA expression in colon samples from WT and *Corin* KO mice. Colon samples were isolated from WT and *Corin* KO mice (male, 10-12 weeks old on normal-salt diet) to extract mRNAs. Quantitative RT-PCR was done to assess *Scnn1a* and *Cftr* mRNA expression levels. n = 6-7 per group. Data are mean ± SEM analyzed by unpaired Student's *t* test.

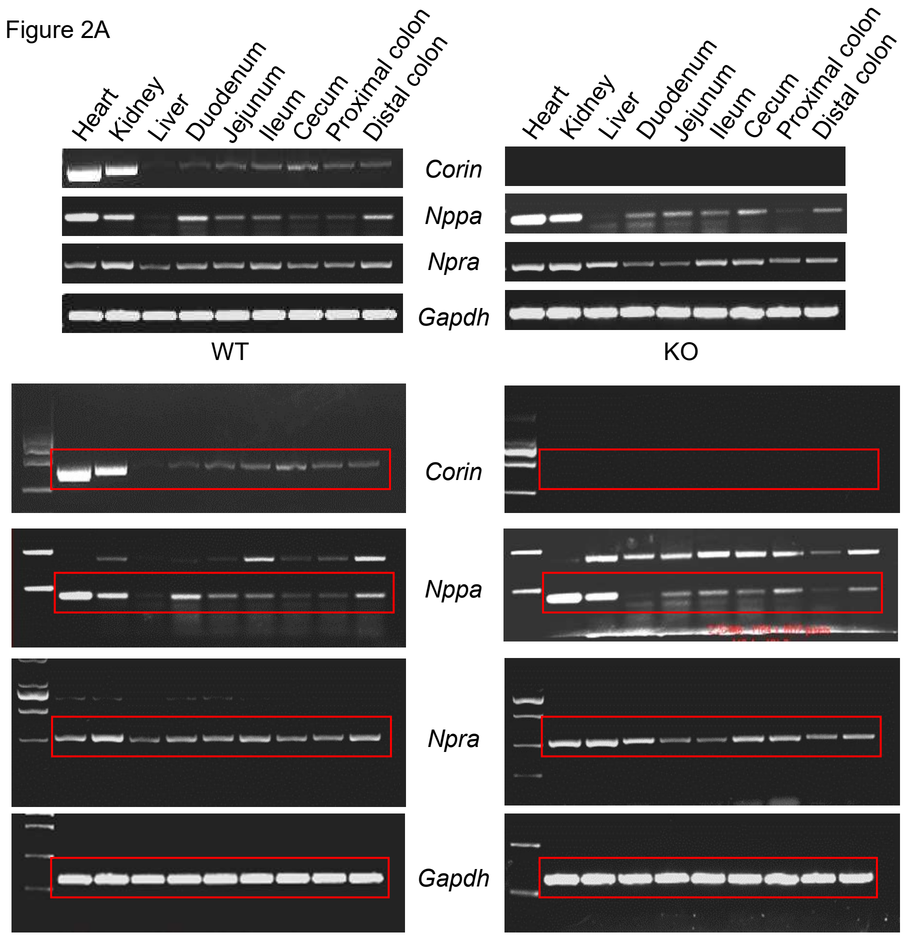

Supplementary Figure S4. Uncropped gel images of Figure 2A.

Table S1. Sequences of oligonucleotide primers used in PCR and RT-PCR

Gene	Locus	Primer	Sequence	Size (bp)
Corin	NM_016869.3	forward	ATCACTCACAGCCAGTGTCAA	390
		reverse	TCCACAGAGTGATTGCTTTCCAT	
Nppa	NM_008725.3	forward	TACAGTGCGGTGTCCAACACAG	126
		reverse	TGCTTCCTCAGTCTGCTCACTC	
Npra	NM_008727.5	forward	GTGTTTGGGCAAAGCCTTCAG	273
		reverse	TGTCACTGCCTGGACATAGA	
Corin	NM_016869.3	forward	CAAGTCTGAGGTCAACTGC	64
		reverse	TGTCCACTTCTGCATTCCAC	
Nppa	NM_008725.3	forward	CACAGATCTGATGGATTTCAAGA	68
		reverse	CCTCATCTTCTACCGGCATC	
Npra	NM_008727.5	forward	GTGTTTGGGCAAAGCCTTCAG	121
		reverse	TGTCACTGCCTGGACATAGA	
Scnn1a	NM_011324.2	forward	TGATGGTGGCTTCAACGTGAGG	97
		reverse	AGTGCAGTCTCCGTAGTTGCCT	
Cftr	NM_021050.2	forward	TGACCTGGAGGAAAACATTAAGA	155
		reverse	AGCCCTGTATGTCTTCACACTG	
Gapdh	NM_001289726.1	forward	TGTTCCTACCCCCAATGTGT	138
		reverse	GGTCCTCAGTGTAGCCCAAG	
